# Contrasting interactions between photon spectra and temperature in cold-sensitive basil and cold-tolerant lettuce

**DOI:** 10.3389/fpls.2025.1675087

**Published:** 2025-09-24

**Authors:** Jiyong Shin, Bruce Bugbee, Erik S. Runkle

**Affiliations:** ^1^ Department of Horticulture, Michigan State University, East Lansing, MI, United States; ^2^ Crop Physiology Laboratory, Utah State University, Logan, UT, United States

**Keywords:** photomorphogenesis, thermomorphogenesis, far-red light, blue light, phytochrome

## Abstract

Blue (B; 400–499 nm) light, far-red (FR; 700–750 nm) light, and temperature are key regulators of plant growth and development, with responses varying by species. While the independent effects of these environmental signals are well established, their interactive effects are not clear. We postulated that the effects of FR light and temperature would depend on the photon flux density (PFD) of B light. To test this, we grew cold-tolerant lettuce and cold-sensitive basil at 19 and 24°C under lighting treatments with three FR fractions [FR-PFD divided by the sum of red (600–699 nm) and FR PFD; 0.01, 0.19, or 0.32] and two B-PFDs (40 or 100 µmol m^−2^ s^−1^). The total PFD (400–750 nm; 270 µmol m^−2^ s^−1^) and photoperiod (24 h d^−1^) were the same in all treatments. There were significant differences between species. As expected, increasing the FR fraction dramatically increased shoot expansion in lettuce and internode elongation in basil. The shoot expansion in lettuce was amplified by higher temperature but attenuated by higher B-PFD. Unlike lettuce, the FR effect on basil internodes did not interact with either temperature or B-PFD. The increased shoot expansion in lettuce decreased foliage coloration, but coloration was minimally altered in basil. These results reveal fundamentally different species responses to light and temperature that may have implications for shade-avoidant and shade-tolerant species. Overall, these findings demonstrate the complex integration of environmental signals in the regulation of growth.

## 1 Introduction

The photon spectrum (or spectral quality), the photon flux density (PFD), and temperature independently regulate plant growth, but their effects can interact and vary among species (Paradiso and Proietti, 2021; Jeong et al., 2024b). A high far-red fraction [far-red (FR; 700–750 nm) PFD/red (R; 600–699 nm)+FR PFD] and a low blue (B; 400–499 nm) PFD (e.g., B-PFDs lower than the ones of unfiltered sunlight) are environmental cues for shading from nearby vegetation. These photon signals induce morphological responses such as leaf, stem, and/or petiole elongation, increased apical dominance, and/or hyponasty (Franklin, 2008; Keuskamp et al., 2010; Keller et al., 2011; Casal, 2013; Gommers et al., 2013; Pedmale et al., 2016; Kusuma and Bugbee, 2021a; Shin and Runkle, 2025; Skabelund et al., 2025). Temperature also regulates the phenotypic and developmental responses of plants, which is known as thermomorphogenesis. Thermomorphogenesis mimics some morphological responses to a high FR fraction and a low B-PFD including stem elongation and leaf hyponasty, although specific responses vary among species (Erwin et al., 1989; Koini et al., 2009; Fukuda et al., 2011; Patel et al., 2013; Snowden et al., 2016; Skabelund et al., 2025).

Photon signals for morphological responses are perceived by plants through several classes of photoreceptors including R and FR photon-absorbing phytochromes and B photon-absorbing cryptochromes (Banerjee and Batschauer, 2005; Chaves et al., 2011; Zhang et al., 2013; Possart et al., 2014). Among several types of phytochrome (PHY), PHYB plays a dominant role in mediating the morphological responses to FR photons in light-grown plants (Sharrock and Clack, 2002). PHYB is homo- or hetero-dimetric chromoproteins that exist in three forms, P_R_P_R_, P_R_P_FR_, and P_FR_P_FR_. The P_R_P_R_ is biologically inactive and is converted to P_R_P_FR_ and P_FR_P_FR_ upon R photon absorption, but is switched back to P_R_P_R_ upon FR photon absorption (Burgie and Vierstra, 2014; Legris et al., 2016; Klose et al., 2020). The phytochrome photoequilibria [PPE; P_FR_/(P_R_+P_FR_)] integrates the effect of photons from 350 to 760 nm and, at least theoretically, provides an improved metric to predict PHY responses (Lagarias et al., 1987; Sager et al., 1988). When the PPE is low, extension growth is induced, and vice versa (Park and Runkle, 2017; Meng and Runkle, 2019). Internal PPE (iPPE), which accounts for spectral distortion within leaves of light-grown plants, has better predicted plant morphological responses than the more original metric of PPE (Kusuma and Bugbee, 2021b).

PHYB functions not only as photoreceptor but also as a thermo-sensor in plants (Jung et al., 2016; Legris et al., 2016; Qiu et al., 2019). An increase in temperature reverts photon-induced P_FR_P_FR_ and P_R_P_FR_ to P_R_P_FR_ and P_R_P_R_, respectively, the rate of which increases exponentially with temperature (Klose et al., 2015, 2020). Therefore, FR fraction and temperature signals can converge at PHYB. Additionally, FR fraction and temperature signaling share downstream pathways (Proveniers and van Zanten, 2013; Qiu et al., 2019). For example, temperature can regulate the expression of the phytochrome-interacting factor (PIF) family, which are targets of active PHYB, through PHYB-independent mechanisms (Castillon et al., 2007; Leivar and Quail, 2011; Raschke et al., 2015; Chung et al., 2020; Jung et al., 2020; Casal and Fankhauser, 2023). Consistent with the signaling convergence, FR photons and temperature interacted to promote hypocotyl elongation in velvetleaf (*Abutilon theophrasti*) and Arabidopsis (*Arabidopsis thaliana*) seedlings (Weinig, 2000; Romero-Montepaone et al., 2020, 2021; Burko et al., 2022). However, given the diverse responsiveness to photon spectra and temperature among model species, whether this interaction between FR photons and temperature is transferable to horticultural crops remains largely unknown. Additionally, it is uncertain whether the responses in seedlings also occur in mature plants. Cryptochromes are flavoproteins that primarily perceive B photon signals (Cashmore et al., 1999). A low B-PFD, sensed by two types of cryptochrome [cryptochrome1 (CRY1) and cryptochrome2 (CRY2)], induces morphological responses including hypocotyl elongation (Ahmad et al., 1995). CRYs activated by B photons can suppress PIFs through physical interaction (Foreman et al., 2011; Ma et al., 2016; Pedmale et al., 2016). Therefore, the interaction between FR photons and temperature can be potentially modulated by B photons. However, this potential influence of B photons has not been considered and merits investigation.

Along with morphology, the photon spectrum and temperature can interact to regulate pigment accumulation, which influences foliage coloration. The biosynthesis of anthocyanins, which is a group of common red- and blue-colored phytopigments, can be triggered by ultraviolet (100–399 nm) and B photons. This process is positively regulated by transcription factors such as the LONG HYPOCOTYL5 (HY5), negatively regulated by CONSTITUTIVE PHOTOMORPHOGENIC1 (COP1), and/or bidirectionally regulated by myeloblastosis (MYB) (Borevitz et al., 2000; Meng and Runkle, 2019; LaFountain and Yuan, 2021). In contrast, high temperature represses anthocyanin biosynthesis by degrading the HY5 protein in a COP1 activity-dependent manner and/or by down-regulating anthocyanin biosynthetic MYB genes (Kim et al., 2017; Rehman et al., 2017). Beyond biosynthesis repression, high temperatures can degrade accumulated anthocyanins (Rehman et al., 2017) and decrease foliage coloration (Tarr et al., 2023). The influence of FR fraction on coloration varies by growth stage, partly because dominant PHY differ by growth stages (Li and Kubota, 2009; Carvalho and Folta, 2014; Li et al., 2014; Liu et al., 2015; Shin and Runkle, 2024). For example, PHYA predominates during seedling de-etiolation, whereas PHYB is dominant in light-grown mature plants. PHY activated either by R or FR photons can suppress COP1 (Sheerin et al., 2015) and can trigger foliage coloration. Given the convergence of photon spectra and air temperature signaling, these environmental factors may interact to regulate the coloration of horticultural crops, particularly those with red- and purple-colored leaves.

The objective of this study was to explore interactions among photon spectra (specifically FR fraction and B-PFD) and air temperature in regulating biomass accumulation, morphological traits, and foliage coloration of two diverse horticultural species that are commonly grown in indoor agriculture, lettuce (*Lactuca sativa*) and basil (*Ocimum basilicum*). Lettuce is a cold-tolerant crop whereas basil is relatively cold sensitive. We hypothesized that FR fraction, B-PFD, and air temperature would interdependently control growth and foliage pigmentation, the magnitude of which would vary among species.

## 2 Materials and methods

### 2.1 The propagation phase

Red oakleaf lettuce ‘Rouxai’ and basil ‘Prospera’ were selected based on their diverse responses to photon spectra and temperature. Leaf expansion in lettuce is generally more sensitive to photon spectra than basil (Meng and Runkle, 2019; Shin and Runkle, 2024), but basil has a higher optimum temperature than lettuce (Walters and Currey, 2019; Tarr et al., 2023).

Seeds of lettuce ‘Rouxai’ (Rijk Zwaan, Salinas, CA, United States) and basil ‘Prospera’ (Johnny’s Selected Seeds, Winslow, ME, United States) were sown into 200-cell (individual plug dimensions 2.5 cm × 2.5 cm × 4.0 cm) Rockwool plugs (Grodan AO Plug 25/40; Grodan, Milton, ON, Canada), one seed per cell, on May 16, 2022, June 14, 2022, and August 7, 2024 for the first, second, and third replication (day 0), respectively. The plugs were presoaked with deionized water adjusted with diluted sulfuric acid (J.Y. Baker Inc., Phillipsburg, NJ, United States) to provide a pH = 4.4–4.5 and electrical conductivity (EC) = 0.03 mS cm^−1^ based on values measured with a pH and electrical conductivity meter (HI9814; Hanna Instruments, Woonsocket, RI, United States).

Germination (day 0–2) and seedling growth (day 3–7) were conducted in a walk-in room of the Controlled Environment Lighting Laboratory (Michigan State University, East Lansing, MI, United States). During the germination stage, seeds sprouted in plug trays covered with transparent plastic humidity domes under a photosynthetic PFD (PPFD; photon flux integral between 400 nm and 700 nm in µmol m^−2^ s^−1^) of 180 µmol m^−2^ s^−1^ for 24 h d^−1^ from warm-white (peak = 639 nm, correlated color temperature = 2700 K) light-emitting diodes (LEDs) (Phytofy RL; OSRAM Opto Semiconductors, Beverley, MA, United States). On day 3, the humidity domes were removed. We measured the air temperature near the middle of the room every 10 s by four thermocouples (0.13-mm type E; Omega Engineering, Inc., Stamford, CT, United States) connected to a data logger (CR1000; Campbell Scientific, Inc., Logan, UT, United States) and logged the hourly average. The air temperature was maintained at 23.3 ± 0.1°C, 23.4 ± 0.1°C, and 22.9 ± 0.3°C (mean ± standard deviation) for the first, second, and third replication, respectively. CO_2_ concentration was measured with a silicon-based nondispersive infrared CO_2_ concentration sensor (GMD20, Vaisala, Helsinki, Finland). The mean CO_2_ concentration was 408.3 ± 18.7 ppm, 402.6 ± 23.5 ppm, and 430.7 ± 35.3 ppm for the first, second, and third replication, respectively. Seedlings were sub-fertigated with deionized water supplemented with a water-soluble fertilizer (12N-4P-16K RO Hydro FeED; JR Peters, Inc., Allentown, PA, United States) and magnesium sulfate (Epsom salt, Pennington Seed, Inc., Madison, GA, United States) at a pH = 5.6 and EC = 1.6 mS cm^−1^. The nutrient solution contained the following (in mg L^−1^): 125 N, 42 P, 167 K, 73 Ca, 49 Mg, 39 S, 1.7 Fe, 0.52 Mn, 0.56 Zn, 0.13 B, 0.47 Cu, and 0.13 Mo.

### 2.2 The production phase

**Figure 1:**
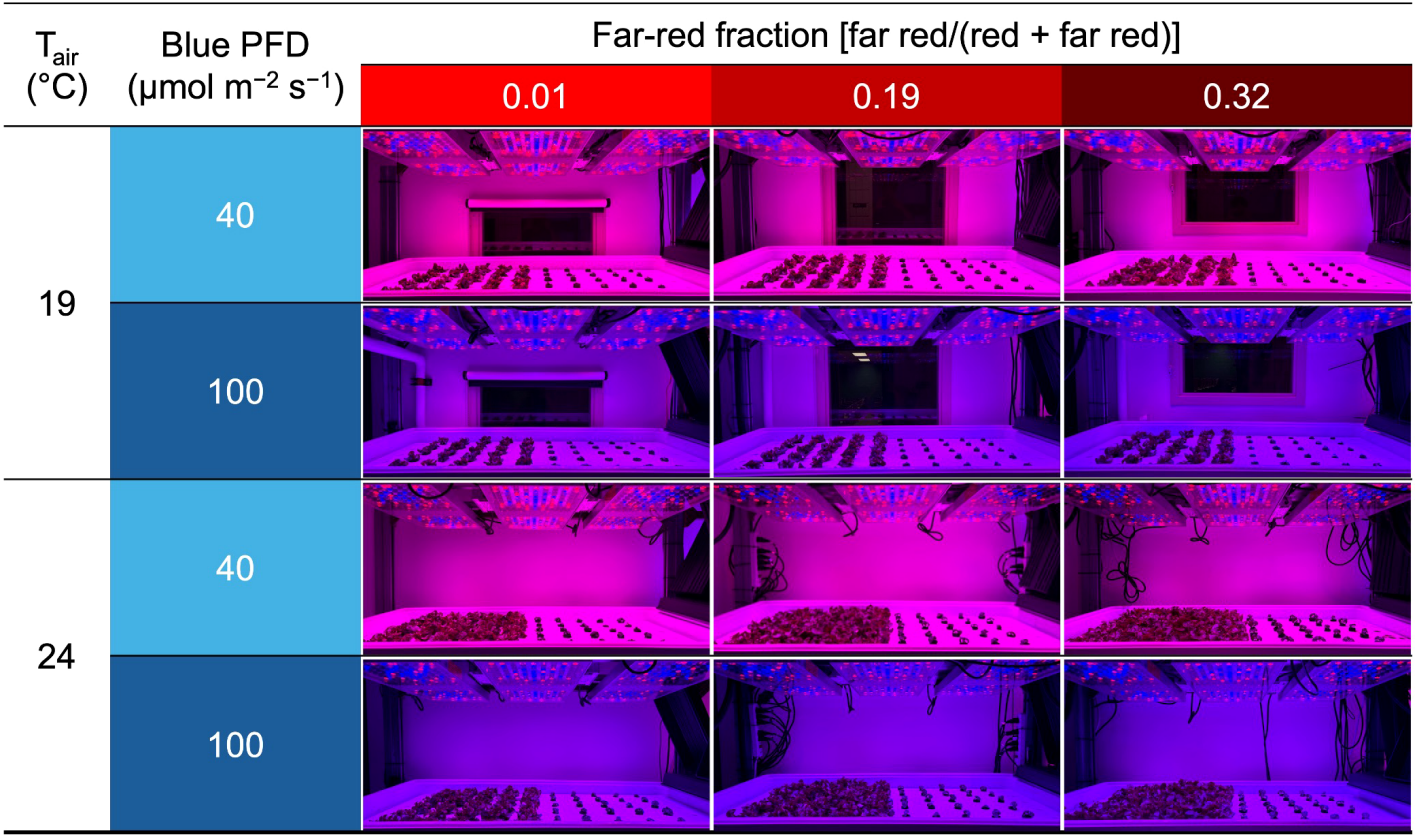
Photographs of the growing system on day 19 (the day of lettuce harvest). T_air_ refers to air temperature. Blue PFD and far-red fraction refer to the blue photon flux density (400–499 nm) and the fraction of the far-red (700–750 nm) photon flux density relative to the sum of the red (600–699 nm) and far-red photon flux density, respectively. Plants on the left and right sides are lettuce ‘Rouxai’ and basil ‘Prospera’, respectively.

On day 8, when the first true leaf of lettuce was expanding and the roots of lettuce and basil reached the base of the plugs, 36 seedlings of each species were transplanted into each of twelve rafts (60.9 cm × 121.9 cm × 2.5 cm raft with 72 holes, each with a diameter of 1 cm; Beaver Plastics, Ltd., Acheson, AB, Canada) floating in deep-flow hydroponic systems in two climate rooms. The plants were spaced 10.0 cm horizontally and 14.1 cm diagonally apart in their respective centers, creating a planting density of 96.8 plants m^−2^ (**Figure 1**). We used the same nutrient solution in the recirculating hydroponic system for the production phase as that used for the propagation phase. Solutions were aerated at 70 L min^−1^ using a round flat air stone (20.3 cm × 2.5 cm; Active Aqua AS8RD; Hydrofarm). During the production phase, CO_2_ concentration remained near the ambient (**Table 1**). The air inside each climate room was continuously circulated to maintain environmental homogeneity.

**Table 1:** Temperature set point (T_air_), actual air temperature (actual T_air_), actual CO_2_ concentration, and average vapor-pressure deficit (VPD) of two air temperature treatments for three experimental replications.

Replication	T_air_ (°C)	Actual T_air_ (°C)	CO_2_ concentration (µmol mol^−1^)	Average VPD (kPa)
First	19	19.0 ± 0.1	415 ± 17	0.7
	24	24.5 ± 0.1	423 ± 21	0.7
Second	19	19.1 ± 0.1	402 ± 20	0.6
	24	24.4 ± 0.1	409 ± 23	0.6
Third	19	19.2 ± 0.7	434 ± 23	0.6
	24	24.5 ± 0.6	449 ± 23	0.9

### 2.3 Temperature and photon spectra treatments

**Figure 2:**
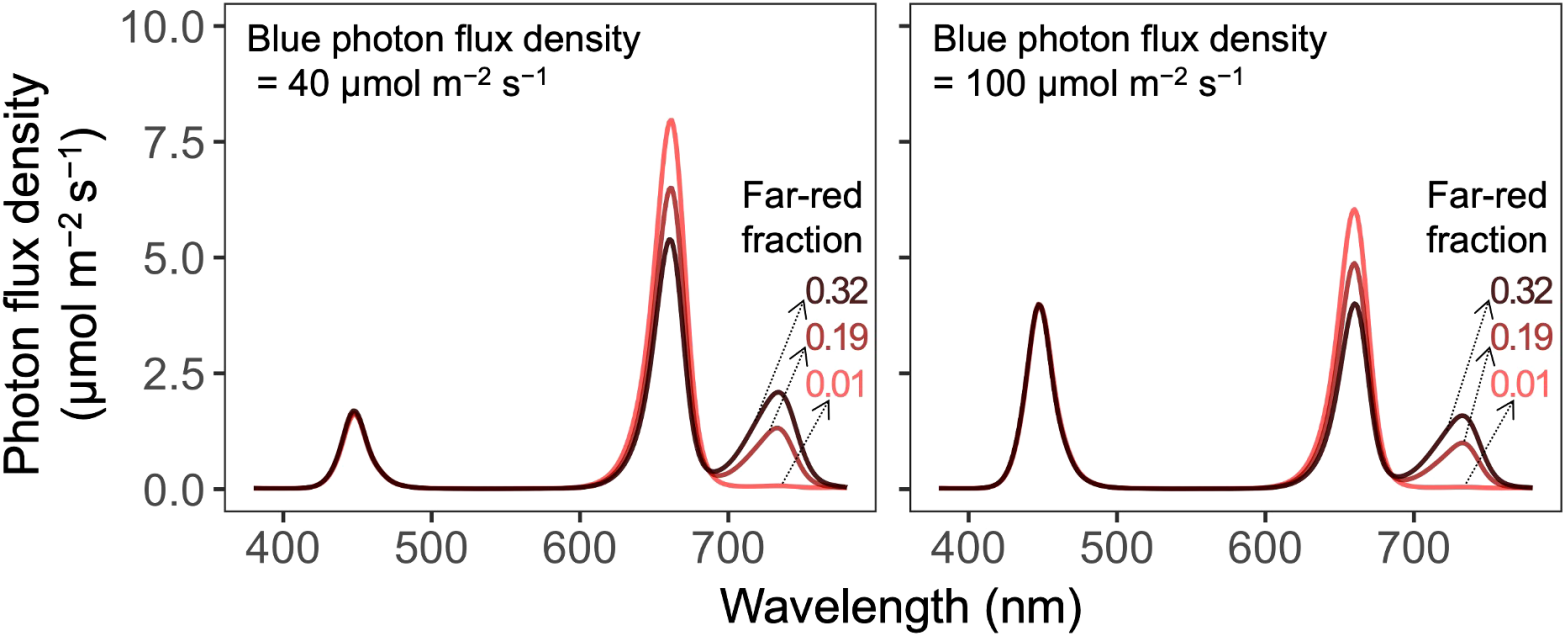
Spectral distributions of six photon spectra treatments with two blue photon flux densities (400–499 nm; in µmol m^−2^ s^−1^) and three far-red fractions [the fraction of far-red (700–750 nm) photon flux density to the sum of red (600–699 nm) and far-red photon flux density] delivered by blue (peak at 447 nm), red (peak at 661 nm), and far-red (peak at 733 nm) light-emitting diodes.

Plants were grown with twelve combinations of the B-PFD (40 or 100 µmol m^−2^ s^−1^), FR fraction (0.01, 0.19, or 0.32), and air temperature (19 or 24°C) (**Tables 1**, **2**). The B-PFD and FR fraction treatments were delivered by the same fixtures as described previously using B, R, and FR narrowband LEDs. The fixtures have dimmable B, R, FR, and warm-white LEDs in the same unit. Photon spectra were measured at twelve locations on the floating raft for each photon spectra treatment using a portable spectroradiometer (LI-180, LI-COR Biosciences, Inc., Lincoln, NE, United States) at initial seedling height (**Figure 2**). As plants developed and grew closer to the LED fixtures, the total PFD (TPFD; photon flux integral between 400 nm and 750 nm in µmol m^−2^ s^−1^) increased by less than 7% (**Supplementary Figure S1**). For each photon spectra treatment, the PPFD, TPFD, R:FR, and estimated iPPE (Kusuma and Bugbee, 2021b) were calculated; the B photon fraction (the B-PFD divided by the TPFD) was 15 or 37%. The TPFD was maintained constant among treatments (270 µmol m^−2^ s^−1^) to minimize differences in photosynthetic photons among treatments. When plants acclimate to FR-enriched conditions, their photosynthetic rate per unit leaf area often decreases with increasing FR fraction under a constant TPFD (Jeong et al., 2024a). This is likely attributable to reduced leaf thickness (i.e., increased specific leaf area; Evans and Poorter, 2001), which can decrease area-based chlorophyll content (Jeong et al., 2024a), a common morphological response to FR photons (Gommers et al., 2013). Nevertheless, several recent studies have demonstrated that FR photons equivalently contribute to photosynthesis when combined with traditionally defined photosynthetically active radiation if the FR-PFD is <30 to 40% of the PPFD (Zhen and Bugbee, 2020; Zhen et al., 2021, 2022; Kelly and Runkle, 2024). This equivalence has been demonstrated both in short-term measurements of unit-area photosynthesis (without acclimation) and in long-term canopy photosynthesis. Consequently, delivering the same TPFD rather than the same PPFD has been increasingly adopted for evaluating the effects of FR photons on plant morphology and growth (Kusuma and Bugbee, 2023; Jeong et al., 2024a, 2024b; Shin and Runkle, 2025; Skabelund et al., 2025). We did not calculate yield photon flux density because it tends to underestimate the contribution of FR photons to photosynthesis (Sager et al., 1988). Lighting was continuous (24 h d^−1^).

**Table 2:** Measured and calculated spectral characteristics of six photon spectra treatments.

Treatment	Photon flux density (µmol m^−2^ s^−1^)					FR fraction^f^	R:FR^g^	iPPE^h^
	Blue^a^	Red^b^	Far red^c^	PAR^d^	Total^e^			
B_40_FRF_0.01_	40	229	3	270	274	0.01	69.4	0.84
B_40_FRF_0.19_	40	184	44	226	270	0.19	4.2	0.58
B_40_FRF_0.32_	42	155	73	198	271	0.32	2.1	0.44
B_100_FRF_0.01_	101	170	2	272	274	0.01	80.9	0.84
B_100_FRF_0.19_	100	137	33	239	272	0.19	4.2	0.58
B_100_FRF_0.32_	100	113	54	214	268	0.32	2.1	0.44

The value following B indicates target blue photon flux density and the value following FRF indicates the far-red fraction of each treatment.

a 400 to 499 nm.

b 600 to 699 nm.

c 700 to 750 nm.

d Photosynthetically Active Radiation: 400 to 700 nm.

e Extended Photosynthetically Active Radiation; ePAR: 400 to 750 nm.

f The fraction of the far-red (700–750 nm) photon flux density (PFD) to the sum of the red (600–699 nm) and far-red PFD.

g The ratio of the red PFD to the far-red PFD.

h Estimated internal phytochrome photoequilibria calculated based on Kusuma and Bugbee (2021b).

The air temperature of each climate room was measured near the middle of each room every 10 seconds by two thermocouples (0.13-mm type E; Omega Engineering, Inc.). The 24°C room was humidified to maintain an atmospheric vapor-pressure deficit (VPD) similar to the 19°C room (**Table 1**). Relative humidity was measured every 10 seconds by a relative humidity probe (HMP110; Vaisala, Inc., Louisville, CO, United States) connected to a data logger (CR1000; Campbell Scientific, Inc) and hourly averages were logged and used for VPD calculation.

### 2.4 Data collection and statistical analysis

Ten lettuce and basil plants per treatment were randomly selected after excluding a few outliers (plants with atypical growth) and were harvested on day 19 and day 32, respectively. Shoots were cut at the surface and dry mass was measured after drying them at 70°C for 5 days in a drying oven (Blue M, Blue Island, IL, United States). The leaf length (mm) of the most expanded leaf was measured. The maximum shoot diameter from a top-down view (for lettuce) and the length of the first internode (for basil) were also measured with a ruler. Area-based chlorophyll concentration was estimated by converting the SPAD values measured by a chlorophyll concentration meter (MC-100, Apogee Instruments, Logan, UT, United States) using generic conversion equations provided by Parry et al. (2014). SPAD values were measured at three locations on the most expanded leaf and were averaged.

To evaluate leaf coloration, the International Commission on Illumination (CIE) L*a*b* values, which indicate darkness-brightness (L*; ranges from 0 to 100), greenness-redness (a*; ranges from −128 to 127), and blueness-yellowness (b*; ranges from −128 to 127), were calculated based on RGB values extracted from images of plants taken overhead using ‘convertColor’ procedure in R statistical analysis software (version 4.1.1; R Foundation for Statistical Computing, Vienna, Austria). Photographs were taken under white fluorescent lamps at an exposure compensation of 0.0, a shutter speed of 1/40 second, and an ISO of 125.

The treatments were assigned in a complete randomized design with three replications in time (*n* = 3). Each response was the mean of ten individual plants per cultivar, but since these plants were in the same nutrient solution, they were not counted as true replicates. Statistical analysis was conducted using R statistical analysis software (version 4.1.1, R Core Team, 2021). The main effects and interaction effects of the treatments were evaluated by type III three-way analysis of variance test. Linear regression was conducted using the ‘lm’ function. Values of *P* < 0.05 were considered statistically significant.

## 3 Results

### 3.1 Shoot extension growth

**Figure 3:**
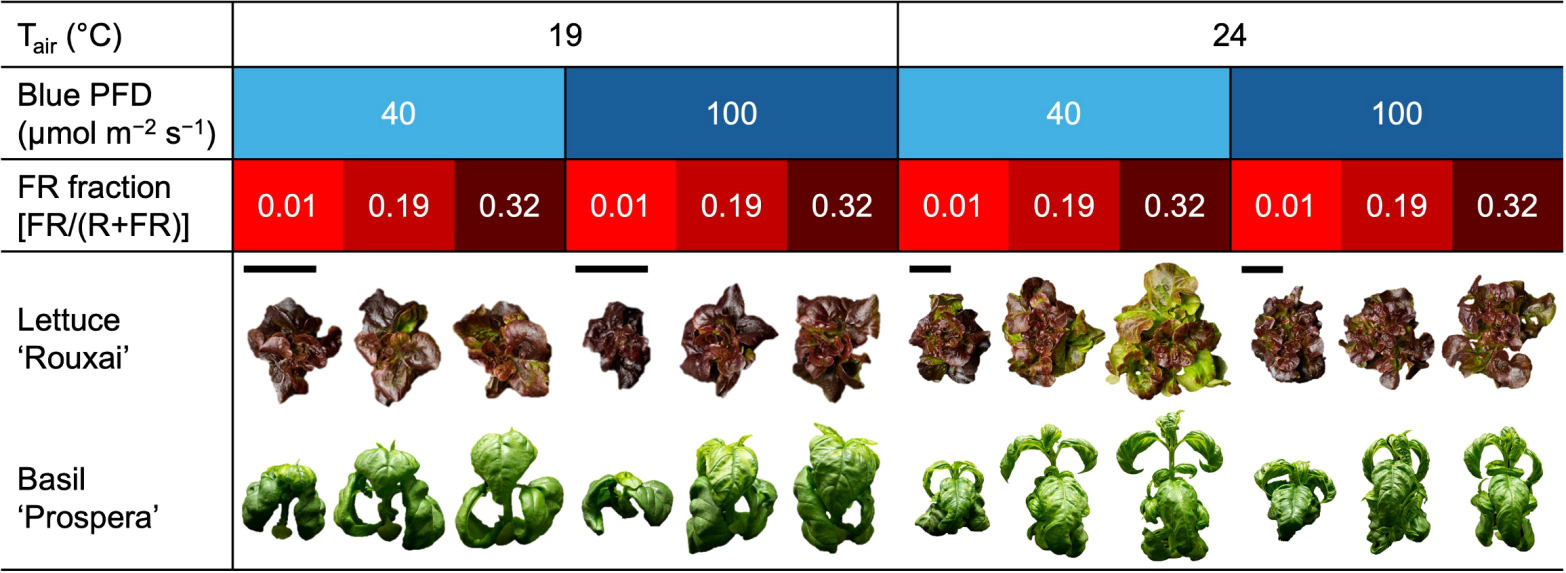
Representative photographs taken 19 or 32 days after seed sow of lettuce ‘Rouxai’ and basil ‘Prospera’, respectively. Plants were grown under six photon spectra treatments with two blue photon flux densities (Blue PFD; 400–499 nm) and three fractions of the far-red (FR; 700–750 nm) photon flux density relative to the sum of the red (R; 600–699 nm) and FR photon flux density (FR fraction) at two air temperatures (T_air_). The size of photographs of plants grown at 19°C was increased by 80% relative to 24°C to improve visibility (bars represent the relative size of photographs).

**Figure 4:**
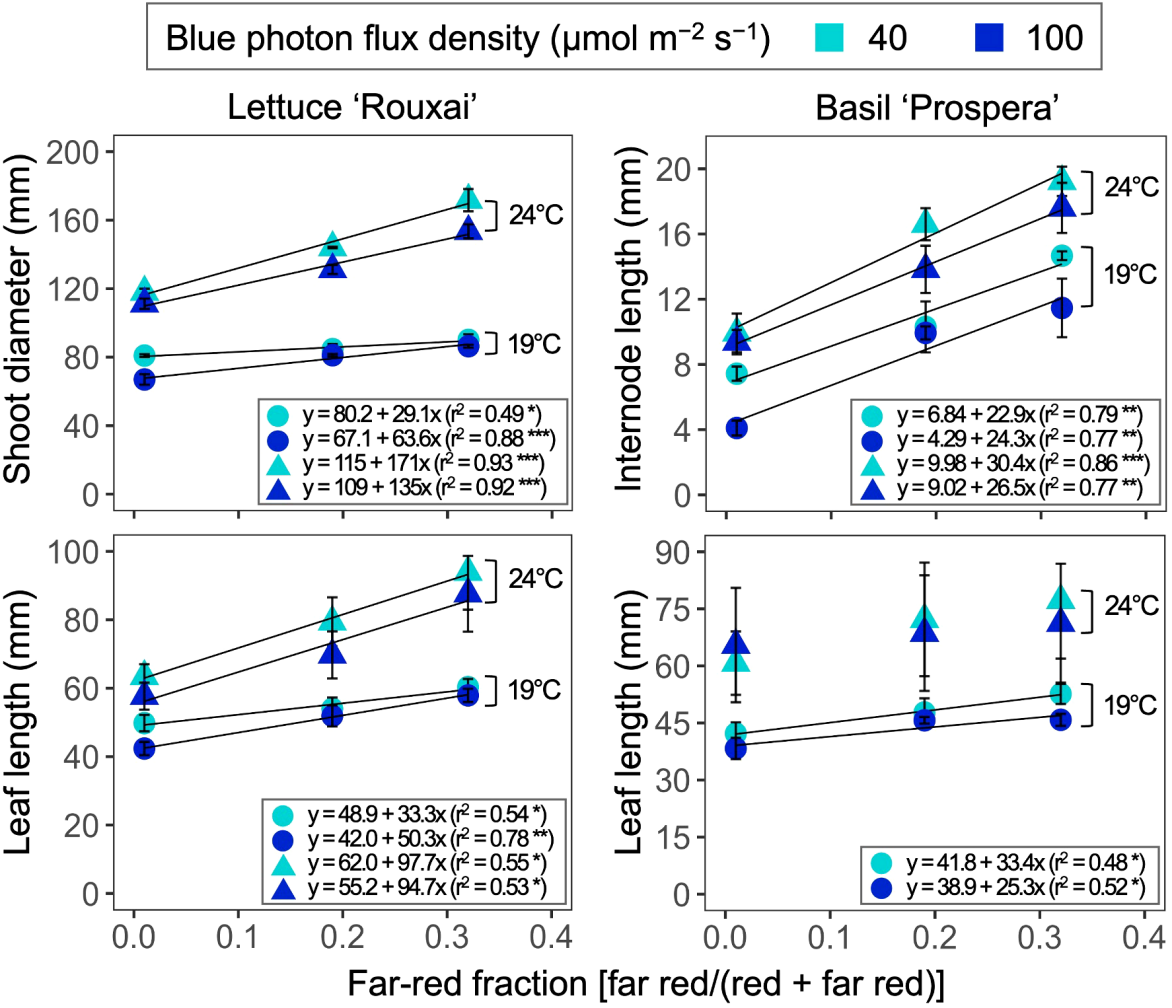
Influence of six photon spectra treatments at two air temperatures on the shoot diameter and leaf length of lettuce ‘Rouxai’ and the internode length and the leaf length of basil ‘Prospera’. The far-red fraction refers to the fraction of the far-red (FR; 700–750 nm) photon flux density relative to the sum of the red (R; 600–699 nm) and FR photon flux density. The unit for blue photon flux density in the legend is µmol m^−2^ s^−1^. Circle (19°C) and triangle (24°C) symbols represent air temperature. Each data point and error bar represents the mean and standard error for three replicates. Regression lines are presented when statistically significant (*P* < 0.05). The coefficient of determination (r^2^) and regression equations are presented for each air temperature and blue photon flux density. *, **, and *** indicate significance at *P* < 0.05, 0.01, or 0.001, respectively.

Increasing the air temperature from 19°C to 24°C caused a 1.3- to 1.9-fold increase in the shoot diameter and leaf length of mature leaves in lettuce (**Figures 3**, **4**, **Table 3**; **Supplementary Figure S1**). Similarly, increasing the FR fraction from 0.01 to 0.32, which decreased the iPPE from 0.84 to 0.44, increased the shoot diameter and the leaf length in lettuce by 1.1 to 1.5 times. The FR-mediated increase in shoot diameter of lettuce was more pronounced at 24°C than at 19°C. An increase in the B-PFD, which generally decreased the shoot diameter of lettuce, decreased the interaction between the FR fraction and air temperature in regulating the shoot diameter (a three-way interaction, *p* = 0.048, **Table 3**; **Supplementary Table S1**). The FR-mediated lettuce leaf elongation was not affected by air temperature.

**Table 3:** Analysis of variance results for the effect of far-red fraction (FRF), blue (B; 400–499 nm) photon flux density, air temperature (T_air_) and their interactions on plant growth and quality parameters of lettuce ‘Rouxai’ and basil ‘Prospera’.

Parameter	Shoot diameter	Internode length	Leaf length	Shoot dry mass	Chlorophyll concentration^a^	L* ^b^	a* ^c^	b* ^d^
Lettuce ‘Rouxai’								
T_air_	***	NA	***	***	***	***	**	***
B	***	NA	NS	**	***	*	*	***
FRF	***	NA	***	*	***	*	NS	***
T_air_ × B	NS	NA	NS	NS	NS	NS	NS	**
T_air_ × FRF	***	NA	NS	NS	NS	NS	*	***
B × FRF	NS	NA	NS	NS	NS	NS	NS	NS
T_air_ × B × FRF	*	NA	NS	NS	NS	NS	NS	NS
Basil ‘Prospera’								
T_air_	NA	***	***	***	***	NS	NS	NS
B	NA	**	NS	NS	NS	*	NS	*
FRF	NA	***	NS	*	***	*	NS	**
T_air_ × B	NA	NS	NS	NS	NS	NS	NS	NS
T_air_ × FRF	NA	NS	NS	NS	NS	NS	NS	NS
B × FRF	NA	NS	NS	NS	NS	NS	NS	NS
T_air_ × B × FRF	NA	NS	NS	NS	NS	NS	NS	NS

NS, *, **, *** indicate non-significance or significance at *P* < 0.05, 0.01, or 0.001, respectively. NA indicates that the growth parameter was not measured.

a Estimated area-based chlorophyll concentration.

b Leaf darkness to brightness using the International Commission on Illumination (CIE) color space.

c Leaf greenness to redness using the CIE color space.

d Leaf blueness to yellowness using the CIE color space.

Increasing the FR fraction increased the internode length by 1.9 to 2.8 times in basil, and the increased air temperature increased the internode length by 1.3 to 2.3 times. These combined effects caused a four-fold increase in internode length (from 4.1 to 17.6 mm). Decreasing the B-PFD caused a smaller but statistically significant increase in internode length (**Figure 4**, **Table 3**; **Supplementary Table S2**). Unlike lettuce, the influence of air temperature, FR fraction, and B-PFD were independent of each other in regulating basil internode length. The FR-fraction effect on basil leaf length was small and was only statistically significant at 19°C.

### 3.2 Shoot biomass and chlorophyll concentration

**Figure 5:**
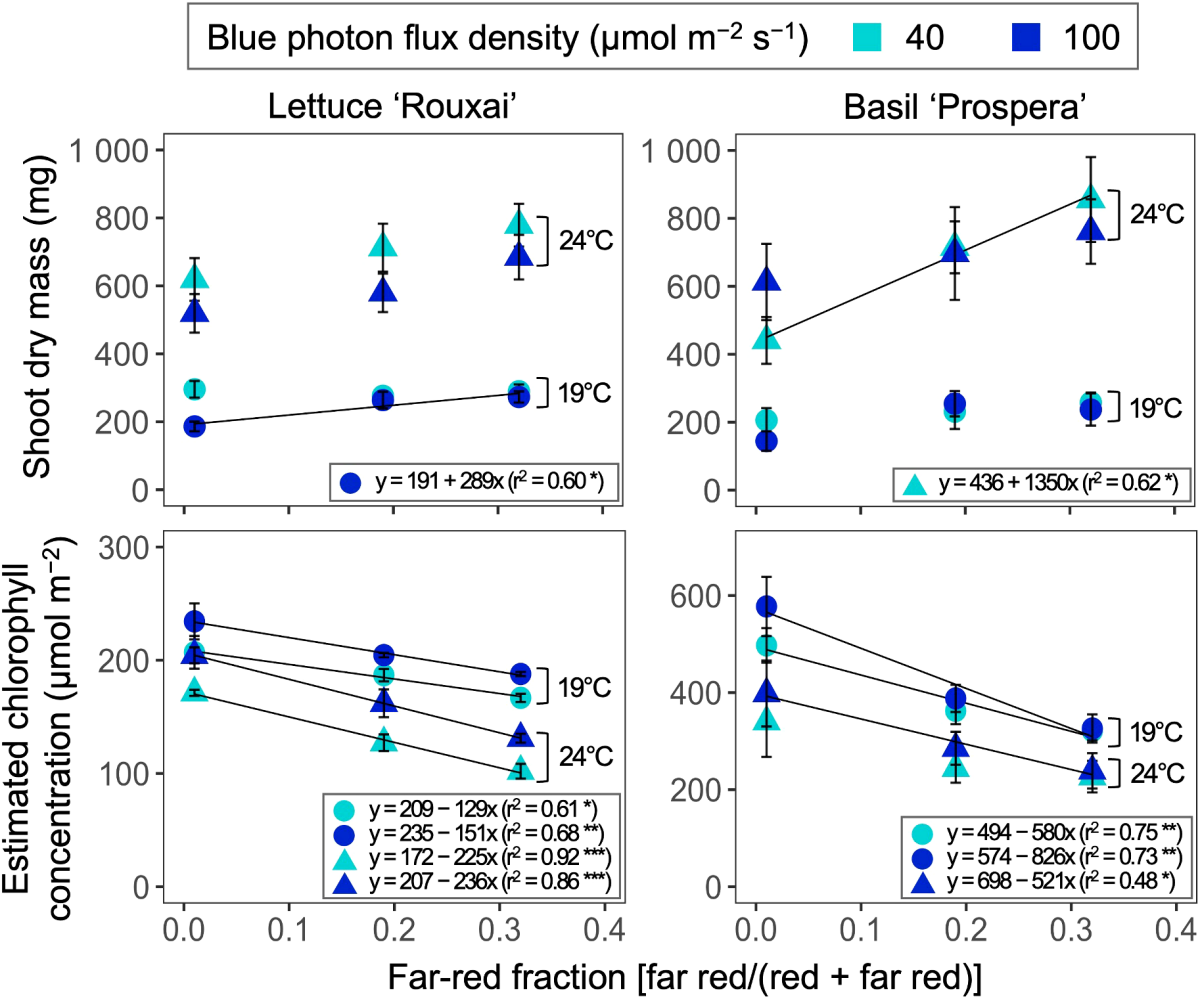
Influence of six photon spectra treatments at two air temperature treatments on the shoot dry mass and estimated area-based chlorophyll concentration of lettuce ‘Rouxai’ and basil ‘Prospera’. The far-red fraction refers to the fraction of the far-red (FR; 700–750 nm) photon flux density relative to the sum of the red (R; 600–699 nm) and FR photon flux density. The unit for blue photon flux density in the legend is µmol m^−2^ s^−1^. Circle (19°C) and triangle (24°C) symbols represent air temperature. Each data point and error bar represents the mean and standard error for three replicates. Regression lines are presented when statistically significant (*P* < 0.05). The coefficient of determination (r^2^) and regression equations are presented for each air temperature and blue photon flux density. *, **, and *** indicate significance at *P* < 0.05, 0.01, or 0.001, respectively.

Increasing the air temperature from 19°C to 24°C increased the shoot dry mass of lettuce by 2.1 to 2.8 times, and the shoot dry mass of basil by 2.1 to 4.3 times (**Tables 3**; **Supplementary Table S1**, **S2**; **Figure 5**). Increasing the FR fraction from 0.01 to 0.32 significantly increased the shoot dry mass of lettuce only at 19°C and under a B-PFD of 100 µmol m^−2^ s^−1^. Overall, within the test range of 19–24°C, the influence of the FR fraction on the shoot dry mass of lettuce was not affected by air temperature. Similarly, increasing the FR fraction increased the shoot dry mass of basil only at 24°C and under the B-PFD of 40 µmol m^−2^ s^−1^; however, air temperature did not influence the effect of the FR fraction on the shoot dry mass. FR fraction did not interact with air temperature and B-PFD to regulate leaf number per plant (data not shown).

Increasing the air temperature and FR fraction consistently decreased the estimated area-based chlorophyll concentration of both species, but B-PFD and air temperature did not influence the FR-mediated decrease in estimated chlorophyll concentration in either species.

### 3.3 Foliage coloration

**Figure 6:**
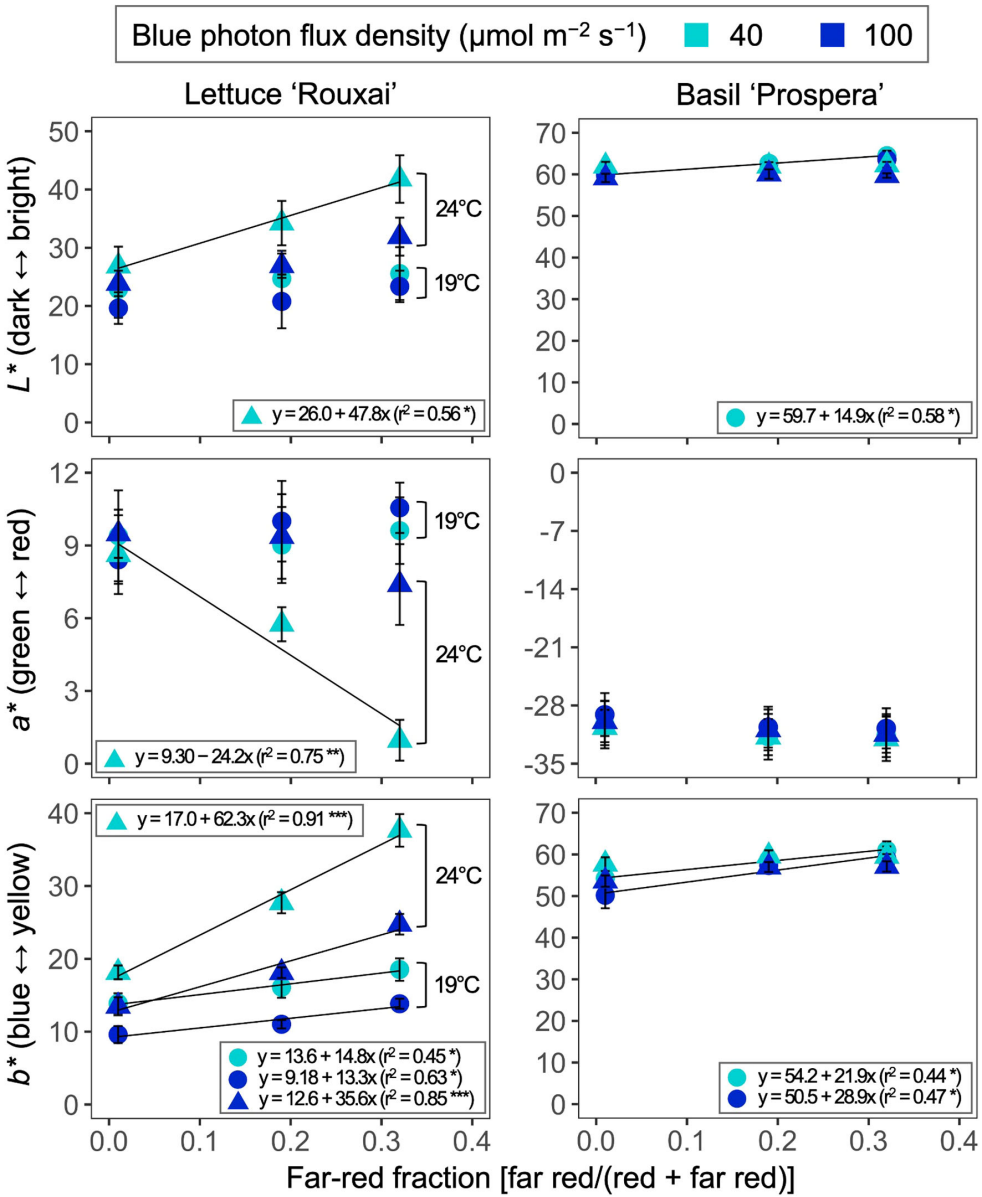
Influence of six photon spectra treatments at two air temperature treatments on CIE L*, a*, and b* of lettuce ‘Rouxai’ and basil ‘Prospera’. L* represents darkness-brightness (from 0 to 100), a* represents greenness-redness (from −128 to 127), and b* represents blueness-yellowness (from −128 to 127) based on the International Commission on Illumination. The far-red fraction refers to the fraction of the far-red (FR; 700–750 nm) photon flux density relative to the sum of the red (R; 600–699 nm) and FR photon flux density. The unit for blue photon flux density in the legend is µmol m^−2^ s^−1^. Circle (19°C) and triangle (24°C) symbols represent air temperature. Each data point and error bar represents the mean and standard error for three replicates. Regression lines are presented when statistically significant (*P* < 0.05). The coefficient of determination (r^2^) and regression equations are presented for each air temperature and blue photon flux density. *, **, and *** indicate significance at *P* < 0.05, 0.01, or 0.001, respectively.

Increasing the air temperature generally increased the leaf brightness (L*) in lettuce (**Figures 2**, **6**, **Table 3**; **Supplementary Table S1**). Increasing the FR fraction from 0.01 to 0.32 significantly increased the brightness of lettuce leaves only at an air temperature of 24°C and under the B-PFD of 40 µmol m^−2^ s^−1^. However, overall, the influence of the FR fraction on the leaf brightness of lettuce was not affected by the air temperature and B-PFD. Compared to lettuce, the leaf brightness of basil was less or not responsive to the air temperature, FR fraction, and B-PFD.

Increasing the FR fraction usually did not influence the leaf redness (a*) of lettuce. However, the increase in the FR fraction decreased the redness of lettuce leaves at 24°C and under the low B-PFD. The air temperature, FR fraction, and B-PFD had little to no effect on the leaf redness of basil. The increase in the air temperature and the FR fraction and the decrease in the B-PFD generally increased the yellowness (b*) of lettuce. The FR-mediated increase in leaf yellowness was more pronounced at 24°C than at 19°C. Compared to lettuce, the leaf yellowness of basil was less or not responsive to the air temperature, FR fraction, and B-PFD.

## 4 Discussion

### 4.1 Mechanisms of the interaction between photon spectra and temperature

Lettuce shoot expansion elicited by an increase in the FR fraction was promoted by an increase in temperature. Similar to our results, the hypocotyl length of velvetleaf was more responsive to the FR fraction (or R:FR) at a day/night temperature of 26/20°C than at 18/16°C (Weinig, 2000). Also, hypocotyl elongation to the FR fraction was more pronounced at warmer temperatures in Arabidopsis (Romero-Montepaone et al., 2020; Burko et al., 2022). A high FR fraction reverts active PHYB to inactive PHYB, which increases the stability of PIFs (Sessa et al., 2005; Hersch et al., 2014). Similarly, high temperature decreases the activity of PHYB, which subsequently releases suppression on PIFs (Gray et al., 1998; Klose et al., 2015; Jung et al., 2016). In addition, high temperature reduces the suppression of PIFs in PHYB-independent manners. For example, high temperatures directly increase PIF7 expression (Chung et al., 2020; Fiorucci et al., 2020; Casal and Fankhauser, 2023). Also, high temperatures enhance PIF4 and PIF5 by downregulating EARLY FLOWERING 3, which is a negative regulator (Box et al., 2015; Raschke et al., 2015; Jung et al., 2020). Greater stability of PIF induced by a high FR fraction, combined with the increased PIF expression triggered by high temperatures, may amplify PIF effects (Romero-Montepaone et al., 2020; Burko et al., 2022; Casal and Fankhauser, 2023). Thus, FR-mediated auxin and gibberellin biosynthesis can be more pronounced at a high temperature, enhancing morphological responses to the FR fraction (Jones and Kaufman, 1983; Evans and Cleland, 1985).

An increase in the B-PFD diminished the interactive effects of the FR fraction and temperature on lettuce shoot expansion. To our knowledge, the modulation of the interaction between the FR fraction and temperature by B-PFD has not been previously reported. Cryptochromes can directly or indirectly suppress PIFs (Foreman et al., 2011; Ma et al., 2016; Pedmale et al., 2016), which is the convergence point of FR fraction and temperature signals (Romero-Montepaone et al., 2020; Burko et al., 2022; Casal and Fankhauser, 2023). Therefore, an increase in the B-PFD likely negates the interaction between the FR fraction and temperature (Castillon et al., 2007). This implies that the B-PFD can be a confounding factor when investigating and interpreting the interaction between the FR fraction and temperature in regulating plant morphology. However, although the three-way interaction among the FR fraction, temperature, and B-PFD was statistically significant in regulating the shoot expansion of lettuce, its biological significance was limited within the B-PFD range tested in our study.

### 4.2 Integrating spectra across multiple wavelengths: effects on internal phytochrome photoequilibria

**Figure 7:**
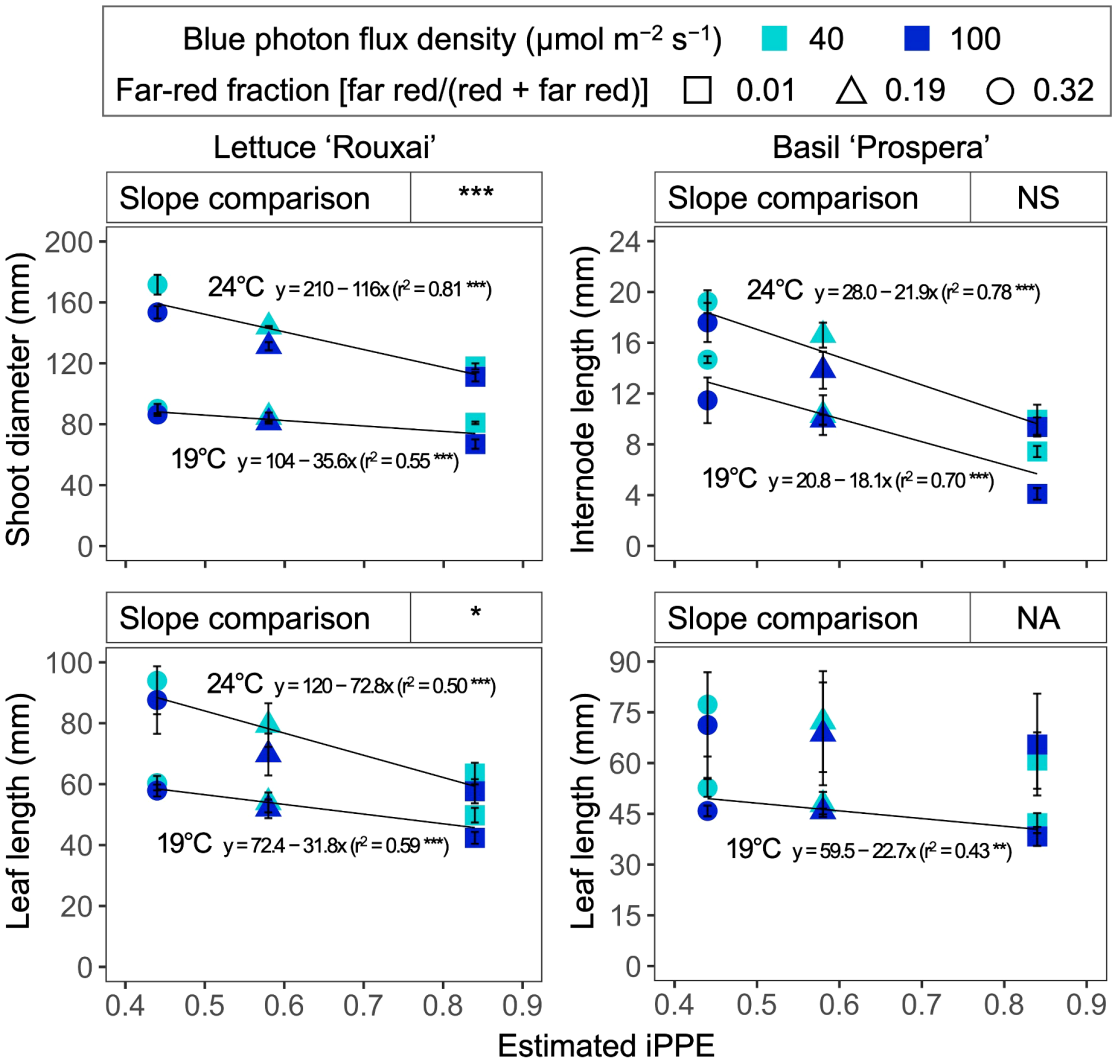
Influence of the estimated internal phytochrome photoequilibria (iPPE; Kusuma and Bugbee, 2021b) of six photon spectra treatments at two air temperature treatments on the shoot diameter and leaf length of lettuce ‘Rouxai’ and on the internode and leaf length of basil ‘Prospera’. The unit for blue (400–499 nm) photon flux density in the legend is µmol m^−2^ s^−1^. FR fraction in the legend refers to the fraction of the far-red (FR; 700–750 nm) photon flux density relative to the sum of the red (R; 600–699 nm) and FR photon flux density. Each data point and error bar represents the mean and standard error for three replicates. Regression lines are presented when statistically significant (*P* < 0.05). The coefficient of determination (r^2^) and regression equations are presented for each air temperature. The slope comparison at the top of each subfigure refers to the difference between the slope of the line regressed for each temperature. NA indicates that the slope comparison was not applicable. NS, *, or *** indicate non-significance or significance at *P* < 0.05 or 0.001, respectively.

The shoot diameter of lettuce and the leaf length of lettuce decreased as iPPE increased from 0.44 to 0.84 (i.e., the FR fraction decreased from 0.32 to 0.01) (**Figure 7**). The absolute slope of the regressed lines for the shoot diameter and leaf length of lettuce was greater when the air temperature was higher. This indicates a greater impact of iPPE on the shoot diameter and leaf length of lettuce at higher air temperatures, and vice versa. In contrast, while the increase in estimated iPPE also decreased the internode length of basil, the slope of the lines regressed for each temperature was similar. This indicates that the influence of iPPE on regulating the internode length of basil is not affected by temperature in the range tested in our study. These response differences between temperatures and plant species suggest that the versatility and power of iPPE in estimating the growth responses of plants can decrease when iPPE is used in a range of temperatures and/or plant species. Similar to these findings, the effects of PPE on the growth and morphology varied among plant species (Park and Runkle, 2017). Also, although specific responses differed, the effects of PPE on the growth and morphology of lettuce and basil varied by air temperature (Jeong et al., 2024a).

### 4.3 The interaction between photon spectra and temperature on foliage coloration

The FR fraction and temperature interacted to regulate leaf redness (i.e., CIE a*) of lettuce ‘Rouxai’. Specifically, lettuce was the most green (least red) at the higher temperature, lower B-PFD, and highest FR fraction, but the effects of FR fraction were greatly diminished at the lower temperature and higher B-PFD. Considering that the responses of a* to the temperature, B-PFD, and FR fraction differed from those of the estimated area-based chlorophyll concentration, the decreases in a* are more likely from a decrease in leaf redness than an increase in leaf greenness. A high temperature represses anthocyanin biosynthesis and facilitates the degradation of accumulated anthocyanins by degrading the HY5 protein in a COP1 activity-dependent manner and by repressing anthocyanin biosynthetic MYB gene expression (Kim et al., 2017; Rehman et al., 2017). The regulation of anthocyanin biosynthesis induced by B photons is also mediated by HY5, COP1, and MYB transcription factors (Borevitz et al., 2000; Meng and Runkle, 2019; LaFountain and Yuan, 2021). Due to the overlap of the regulatory pathway, the high temperature in this study might have amplified the effects of a low B-PFD in decreasing the anthocyanin concentration and leaf redness. Also, amplified extension growth by a high FR fraction and a low B-PFD at the high air temperature might have diluted the anthocyanin content, thus decreasing the anthocyanin concentration and leaf coloration.

Although foliage coloration can provide proxies of pigment concentration, it does not always perfectly align with the concentration of individual pigments (Kelly and Runkle, 2023). This is partly because foliage color is determined by the combined effects of multiple pigments, which can mask one another. Thus, the interpretation of a* values in this study should be considered an indirect estimation of pigmentation. Similarly, as we estimated chlorophyll concentration through SPAD measurement (Parry et al., 2014), direct pigment extraction and measurement would be needed to confirm the interpretations.

### 4.4 Ecological implications of the interaction between spectral quality and temperature

An increase in temperature increased the responsiveness to FR light in cold-tolerant lettuce but not in cold-sensitive basil. Ecologically, the synergistic interaction between FR photons and temperature in inducing extension growth has been proposed as a morphological acclimation to enhance light capture under shaded conditions, particularly when respiratory demand is high (e.g., at elevated temperatures) (Romero-Montepaone et al., 2021; Casal and Fankhauser, 2023). This interaction between FR photons and temperature has been reported primarily in cold-tolerant plants (i.e., those with a low base temperature) such as Arabidopsis and lettuce (Jung et al., 2016; Romero-Montepaone et al., 2020, 2021; Burko et al., 2022; Jeong et al., 2024b) and is apparently uncommon in cold-sensitive plants (Weinig, 2000). Plants acclimated or adapted to high temperatures generally exhibit low respiration rates and thus have a higher optimum temperature for photosynthesis (Berry and Bjorkman, 1980; Hikosaka et al., 2006; Silim et al., 2010; Wataru et al., 2014). This suggests that cold-sensitive plants would exhibit a smaller increase in carbon demand in response to the same rise in temperature compared with cold-tolerant plants. Therefore, the interaction between FR photons and temperature may be less likely in cold-sensitive crops than in tolerant crops.

The contrasting responses of lettuce (leaf expansion) and basil (stem elongation) to simulated shade conditions (low B-PFD, high FR fraction, or both) and temperature may also be representative of shade-avoidant and shade-tolerant species. These response categories were recently reviewed by Xu et al. (2021) and Casal and Fankhauser (2023), and the implications for breeding high-density soybean genotypes were reviewed by Lyu et al. (2023). The difference might also be linked to the breeding history of lettuce, which focused on extending the harvest window and increasing yield through delaying bolting, during which notable stem elongation occurs (Han et al., 2021). Further investigation with additional cold-sensitive/cold-tolerant plants and shade-avoidant and shade-tolerant plants is needed to determine whether the differences between lettuce and basil in this study are fundamental temperature or shade responses or merely species-specific variation.

The lack of interaction between temperature and the FR fraction in inducing morphological changes in basil has been associated with reduced regulation of basil morphology by the photon spectrum (Meng and Runkle, 2019) relative to other species such as lettuce and Arabidopsis. The plasticity of responses to photon spectra (including PHYB activity and phenotype) can depend on population origin (Ikeda et al., 2021). Species endemic to lower latitudes (e.g., *Cardamine nipponica*) are sometimes less sensitive to R and FR photons than species of the same genus but endemic to higher latitudes (e.g., *Cardamine bellidifolia*) due to innate lower P_FR_ stability of PHYB in the lower-latitude species. The shorter twilight period (when there is a meaningful R:FR change) at lower latitudes than at higher latitudes could have contributed to such adaptation of a PHYB response. Basil originates from sub-tropical and tropical regions (i.e., low latitudes), which have a relatively short twilight period and could have a lower P_FR_ stability and functionality, which can decrease the sensitivity to the FR fraction. Considering that the synergism between the FR fraction and temperature can potentially occur due to the combination of FR-mediated PIF stability increase and temperature-mediated PIF expression increase (Romero-Montepaone et al., 2020; Burko et al., 2022; Casal and Fankhauser, 2023), the innate low sensitivity of basil to the FR fraction might have caused less of an FR-mediated PIF stability increase to make the synergism weaker. Similar to our results, the plant height increase elicited by the FR fraction was similar at 20°C and 24°C in basil ‘Genovese’ (Jeong et al., 2024a).

An elevated B-PFD mitigated the interactive effect between the FR fraction and temperature in regulating lettuce shoot expansion. An increase in B-PFD is an environmental cue indicating sun exposure and high carbon availability (Foreman et al., 2011; Ma et al., 2016; Pedmale et al., 2016). Therefore, our findings suggest that increased B-PFD can function to reduce excessive resource allocation towards extension growth caused by the interaction between the FR fraction and temperature in some cold-tolerant plants such as lettuce.

## 5 Conclusions

Photon spectra (FR fraction and B-PFD) and air temperature interactively regulated the morphology and foliage coloration of lettuce but not basil. Specifically, a high air temperature amplified the effects of a high FR fraction in inducing shoot expansion in lettuce. An increase in the B-PFD, which did not directly interact with air temperature in the range tested in our study, negated the interaction between the FR fraction and temperature. This suggests that the B-PFD can confound the interpretation of the synergism between the FR fraction and temperature in eliciting morphological responses. Such interaction between the FR fraction, B-PFD, and temperature did not occur in basil, which not surprisingly, indicates species-specific variation in morphological responses to the environment.

## Data Availability

Biometric and environmental data are available at https://doi.org/10.15482/USDA.ADC/28121552.
