# Supplementary material for: Contrasting interactions between photon spectra and temperature in cold-sensitive basil and cold-tolerant lettuce
Source: Front Plant Sci. 2025 Sep 24;16:1675087. doi: 10.3389/fpls.2025.1675087 (PMC12504075; doi:10.3389/fpls.2025.1675087)
Supplement: Supplementary file 1 [file Table1.docx]

Supplementary Material


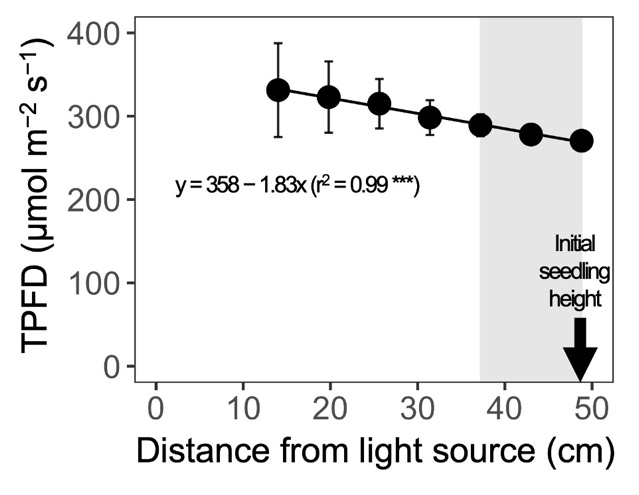


**Supplementary Figure 1.** Total photon flux density (TPFD; 400–750 nm) gradient created by the distance from lighting sources. Each data point and error bar represents the mean and standard error for the TPFD measured at twelve horizontal locations at each distance. The shaded area represents the general range of distance between the light source and plants in this study. The coefficient of determination (r^2^) and regression equations are presented. *** indicates significance at *P* < 0.001.

**Supplementary Table 1.** Full analysis of variance test results for the effect of the fraction of the far-red (FR; 700–750 nm) photon flux density relative to the sum of the red (R; 600–699 nm) and FR photon flux density (FRF), blue (B; 400–499 nm) photon flux density, air temperature (Tair) and their interactions on plant growth and quality parameters of lettuce ‘Rouxai’. NS, *, **, or *** indicate non-significance or significance at *P* < 0.05, 0.01, or 0.001, respectively. SS and DF refer to sums of squares and degree of freedom, respectively.

|  | SS | DF | F value | Pr(>F) |  |
| --- | --- | --- | --- | --- | --- |
| Shoot diameter (mm) | | | | | |
| (Intercept) | 435380 | 1 | 1580 | <0.001 | *** |
| T_air_ | 28798 | 1 | 1040 | <0.001 | *** |
| B | 874 | 1 | 31.8 | <0.001 | *** |
| FRF | 5820 | 2 | 106 | <0.001 | *** |
| T_air_ × B | 69 | 1 | 2.50 | 0.127 | NS |
| T_air_ × FRF | 1717 | 2 | 31.2 | <0.001 | *** |
| B × FRF | 13 | 2 | 0.229 | 0.797 | NS |
| T_air_ × B × FRF | 190 | 2 | 3.45 | 0.048 | * |
| Residuals | 661 | 24 |  |  |  |
| Leaf length (mm) | | | | | |
| (Intercept) | 147494 | 1 | 1440 | <0.001 | *** |
| T_air_ | 4590 | 1 | 44.8 | <0.001 | *** |
| B | 285 | 1 | 2.78 | 0.108 | NS |
| FRF | 2805 | 2 | 13.7 | <0.001 | *** |
| T_air_ × B | 24 | 1 | 0.236 | 0.631 | NS |
| T_air_ × FRF | 446 | 2 | 2.18 | 0.135 | NS |
| B × FRF | 8 | 2 | 0.0380 | 0.963 | NS |
| T_air_ × B × FRF | 32 | 2 | 0.157 | 0.856 | NS |
| Residuals | 2457 | 24 |  |  |  |
| Shoot dry mass (g) | | | | | |
| (Intercept) | 7501893 | 1 | 1160 | <0.001 | *** |
| T_air_ | 1332512 | 1 | 206 | <0.001 | *** |
| B | 54152 | 1 | 8.36 | 0.008 | ** |
| FRF | 61932 | 2 | 4.78 | 0.018 | * |
| T_air_ × B | 8813 | 1 | 1.36 | 0.255 | NS |
| T_air_ × FRF | 22739 | 2 | 1.76 | 0.194 | NS |
| B × FRF | 3715 | 2 | 0.287 | 0.753 | NS |
| T_air_ × B × FRF | 6562 | 2 | 0.507 | 0.609 | NS |
| Residuals | 155451 | 24 |  |  |  |
| Chlorophyll concentration (µmol m^−2^) | | | | | |
| (Intercept) | 1086741 | 1 | 522 | <0.001 | *** |
| T_air_ | 20968 | 1 | 101 | <0.001 | *** |
| B | 6662 | 1 | 32.0 | <0.001 | *** |
| FRF | 19922 | 2 | 47.9 | <0.001 | *** |
| T_air_ × B | 241 | 1 | 1.16 | 0.293 | NS |
| T_air_ × FRF | 1202 | 2 | 2.89 | 0.075 | NS |
| B × FRF | 43 | 2 | 0.104 | 0.902 | NS |
| T_air_ × B × FRF | 53 | 2 | 0.128 | 0.880 | NS |
| Residuals | 4993 | 24 |  |  |  |
| CIE *L** | | | | | |
| (Intercept) | 25989 | 1 | 629 | <0.001 | *** |
| T_air_ | 592 | 1 | 14.3 | <0.001 | *** |
| B | 217 | 1 | 5.27 | 0.031 | * |
| FRF | 325 | 2 | 3.93 | 0.033 | * |
| T_air_ × B | 29 | 1 | 0.709 | 0.408 | NS |
| T_air_ × FRF | 103 | 2 | 1.25 | 0.304 | NS |
| B × FRF | 15 | 2 | 0.184 | 0.833 | NS |
| T_air_ × B × FRF | 24 | 2 | 0.289 | 0.752 | NS |
| Residuals | 991 | 24 |  |  |  |
| CIE *a** | | | | | |
| (Intercept) | 2429 | 1 | 416 | <0.001 | *** |
| T_air_ | 59 | 1 | 10.2 | 0.004 | ** |
| B | 35 | 1 | 6.00 | 0.022 | * |
| FRF | 22 | 2 | 1.92 | 0.169 | NS |
| T_air_ × B | 25 | 1 | 4.26 | 0.050 | NS |
| T_air_ × FRF | 57 | 2 | 4.86 | 0.017 | * |
| B × FRF | 22 | 2 | 1.85 | 0.179 | NS |
| T_air_ × B × FRF | 5 | 2 | 0.469 | 0.631 | NS |
| Residuals | 140 | 24 |  |  |  |
| CIE *b** | | | | | |
| (Intercept) | 12406 | 1 | 2410 | <0.0001 | *** |
| T_air_ | 807 | 1 | 157 | <0.001 | *** |
| B | 424 | 1 | 82.4 | <0.001 | *** |
| FRF | 591 | 2 | 57.4 | <0.001 | *** |
| T_air_ × B | 43 | 1 | 8.30 | 0.008 | ** |
| T_air_ × FRF | 180 | 2 | 17.5 | <0.001 | *** |
| B × FRF | 29 | 2 | 2.83 | 0.079 | NS |
| T_air_ × B × FRF | 23 | 2 | 2.24 | 0.129 | NS |
| Residuals | 124 | 24 |  |  |  |

**Supplementary Table 2.** Full analysis of variance test results for the effect of the fraction of the far-red (FR; 700–750 nm) photon flux density relative to the sum of the red (R; 600–699 nm) and FR photon flux density (FRF), blue (B; 400–499 nm) photon flux density, air temperature (Tair) and their interactions on plant growth and quality parameters of basil ‘Prospera’. NS, *, **, or *** indicate non-significance or significance at *P* < 0.05, 0.01, or 0.001, respectively. SS and DF refer to sums of squares and degree of freedom, respectively.

|  | SS^a^ | DF^b^ | F value | Pr(>F) |  | |
| --- | --- | --- | --- | --- | --- | --- |
| Internode length (mm) | | | | | |  |
| (Intercept) | 5218 | 1 | 1440 | <0.001 | *** | |
| T_air_ | 205 | 1 | 56.6 | <0.001 | *** | |
| B | 35 | 1 | 9.70 | 0.005 | ** | |
| FRF | 394 | 2 | 54.3 | <0.001 | *** | |
| T_air_ × B | 1 | 1 | 0.257 | 0.617 | NS | |
| T_air_ × FRF | 4 | 2 | 0.509 | 0.608 | NS | |
| B × FRF | 1 | 2 | 0.150 | 0.862 | NS | |
| T_air_ × B × FRF | 11 | 2 | 1.51 | 0.241 | NS | |
| Residuals | 88 | 24 |  |  |  | |
| Leaf length (mm) | | | | | |  |
| (Intercept) | 118347 | 1 | 373 | <0.001 | *** | |
| T_air_ | 5119 | 1 | 16.1 | <0.001 | *** | |
| B | 78 | 1 | 0.247 | 0.624 | NS | |
| FRF | 635 | 2 | 1.00 | 0.383 | NS | |
| T_air_ × B | 16 | 1 | 0.0491 | 0.826 | NS | |
| T_air_ × FRF | 7 | 2 | 0.0114 | 0.989 | NS | |
| B × FRF | 70 | 2 | 0.110 | 0.896 | NS | |
| T_air_ × B × FRF | 43 | 2 | 0.0676 | 0.935 | NS | |
| Residuals | 7619 | 24 |  |  |  | |
| Shoot dry mass (g) | | | | | |  |
| (Intercept) | 7323293 | 1 | 388 | <0.001 | *** | |
| T_air_ | 1891271 | 1 | 100 | <0.001 | *** | |
| B | 0 | 1 | 0.000 | 0.998 | NS | |
| FRF | 198132 | 2 | 5.24 | 0.013 | * | |
| T_air_ × B | 3527 | 1 | 0.187 | 0.670 | NS | |
| T_air_ × FRF | 65573 | 2 | 1.73 | 0.198 | NS | |
| B × FRF | 19123 | 2 | 0.503 | 0.609 | NS | |
| T_air_ × B × FRF | 42646 | 2 | 1.13 | 0.340 | NS | |
| Residuals | 453555 | 24 |  |  |  | |
| Chlorophyll concentration (µmol m^−2^) | | | | | |  |
| (Intercept) | 4419965 | 1 | 802 | <0.001 | *** | |
| T_air_ | 136596 | 1 | 24.8 | <0.001 | *** | |
| B | 12321 | 1 | 2.24 | 0.148 | NS | |
| FRF | 200742 | 2 | 18.2 | <0.001 | *** | |
| T_air_ × B | 0 | 1 | 0.000 | 0.999 | NS | |
| T_air_ × FRF | 9766 | 2 | 0.886 | 0.425 | NS | |
| B × FRF | 5576 | 2 | 0.506 | 0.609 | NS | |
| T_air_ × B × FRF | 587 | 2 | 0.053 | 0.948 | NS | |
| Residuals | 132206 | 24 |  |  |  | |
| CIE *L** | | | | | |  |
| (Intercept) | 135631 | 1 | 29900 | <0.001 | *** | |
| T_air_ | 10 | 1 | 2.23 | 0.148 | NS | |
| B | 24 | 1 | 5.29 | 0.031 | * | |
| FRF | 33 | 2 | 3.61 | 0.043 | * | |
| T_air_ × B | 6 | 1 | 1.24 | 0.277 | NS | |
| T_air_ × FRF | 21 | 2 | 2.36 | 0.116 | NS | |
| B × FRF | 0 | 2 | 0.0150 | 0.985 | NS | |
| T_air_ × B × FRF | 2 | 2 | 0.223 | 0.802 | NS | |
| Residuals | 109 | 24 |  |  |  | |
| CIE *a** | | | | | |  |
| (Intercept) | 34402 | 1 | 1850 | <0.001 | *** | |
| T_air_ | 2 | 1 | 0.0985 | 0.756 | NS | |
| B | 7 | 1 | 0.385 | 0.541 | NS | |
| FRF | 15 | 2 | 0.411 | 0.668 | NS | |
| T_air_ × B | 0 | 1 | 0.0163 | 0.899 | NS | |
| T_air_ × FRF | 0 | 2 | 0.0089 | 0.991 | NS | |
| B × FRF | 0 | 2 | 0.0044 | 0.996 | NS | |
| T_air_ × B × FRF | 0 | 2 | 0.0004 | ≈1.000 | NS | |
| Residuals | 446 | 24 |  |  |  | |
| CIE *b** | | | | | |  |
| (Intercept) | 117225 | 1 | 10300 | <0.001 | *** | |
| T_air_ | 4 | 1 | 0.377 | 0.545 | NS | |
| B | 69 | 1 | 6.09 | 0.021 | * | |
| FRF | 184 | 2 | 8.12 | 0.002 | ** | |
| T_air_ × B | 1 | 1 | 0.0570 | 0.813 | NS | |
| T_air_ × FRF | 39 | 2 | 1.71 | 0.202 | NS | |
| B × FRF | 8 | 2 | 0.330 | 0.722 | NS | |
| T_air_ × B × FRF | 1 | 2 | 0.0264 | 0.974 | NS | |
| Residuals | 272 | 24 |  |  |  | |
